# The Relationship between Serum 25-Hydroxyvitamin D Concentration, Cardiorespiratory Fitness, and Insulin Resistance in Japanese Men

**DOI:** 10.3390/nu7010091

**Published:** 2014-12-29

**Authors:** Xiaomin Sun, Zhen-Bo Cao, Kumpei Tanisawa, Tomoko Ito, Satomi Oshima, Mitsuru Higuchi

**Affiliations:** 1 Graduate School of Sport Sciences, Waseda University, Tokorozawa, Saitama 359-1192, Japan; E-Mails: gzhtxiaomin@ruri.waseda.jp (X.S.); kunpei-tanisawa@fuji.waseda.jp (K.T.); maruto7691@moegi.waseda.jp (T.I.); 2 School of Kinesiology, Shanghai University of Sport, 399 Chang Hai Road, Shanghai 200438, China; 3 Faculty of Sport Sciences, Waseda University, Tokorozawa, Saitama 359-1192, Japan; E-Mails: satomioshima@fuji.waseda.jp (S.O.); mhiguchi@waseda.jp (M.H.)

**Keywords:** vitamin D, cardiorespiratory fitness, insulin resistance, visceral fat

## Abstract

Here, we aim to investigate the independent and combined associations of serum 25-hydroxyvitamin D (25(OH)D) and cardiorespiratory fitness (CRF) with glucose metabolism. Fasting blood samples of 107 men aged 40–79 years were analyzed for 25(OH)D, glucose, insulin, glycated hemoglobin, and lipid profile. Homeostasis model assessment of insulin resistance index (HOMA-IR) was calculated from the fasting concentrations of glucose and insulin. Visceral fat area (VFA) was determined by magnetic resonance imaging and CRF by measuring maximal oxygen uptake. Median 25(OH)D concentration was 36.3 nmol/L, while the prevalence of 25(OH)D deficiency was 74.8%. Participants with high CRF had significantly lower HOMA-IR, glycated hemoglobin, and insulin values than participants with low CRF (*p* < 0.05). Higher 25(OH)D concentration was strongly correlated with lower HOMA-IR and insulin values independent of VFA (*p* < 0.01) but significantly affected by CRF. In the high CRF group, participants with higher 25(OH)D concentration had lower HOMA-IR values than participants with low 25(OH)D concentration (*p* < 0.05). Higher 25(OH)D and CRF are crucial for reducing insulin resistance regardless of abdominal fat. In addition, higher 25(OH)D concentration may strengthen the effect of CRF on reducing insulin resistance in middle-aged and elderly Japanese men with high CRF.

## 1. Introduction

Obesity and overweight are the most important risk factors for insulin resistance and type 2 diabetes mellitus (T2DM) [1,2]. In contrast to Caucasians with T2DM who always have high body mass indices (BMI), the Japanese population tends to develop T2DM with low BMI [3,4]. Therefore, besides obesity, other lifestyle factors, such as regular physical activity or ensuring sufficient micronutrient intake, may play an important role in the prevention of insulin resistance or T2DM in Japan.

Vitamin D is of vital importance for bone health and also appears to have extra-skeletal effects [5,6,7,8]. Recent evidence showed that high circulating 25-hydroxyvitamin D (25(OH)D) was associated with low prevalence of increased homeostasis model assessment of insulin resistance (HOMA-IR) and T2DM not only in individuals with impaired glucose tolerance or T2DM [9,10], but also in healthy individuals [11,12,13,14]; these associations were independent of BMI. In addition, several studies documented an association between high cardiorespiratory fitness (CRF) and low prevalence of insulin resistance and T2DM in adults, even after adjustment for BMI [15,16,17,18,19,20,21,22]. However, the possible interaction between circulating 25(OH)D and CRF with regard to insulin resistance has not been studied.

Therefore, the purpose of this study was to investigate the independent and combined associations of serum 25(OH)D and CRF levels with insulin resistance in middle-aged and elderly Japanese men.

## 2. Materials and Methods

### 2.1. Subjects

One hundred and seven Japanese men aged 40–79 years participated in this study. All procedures were conducted in Tokorozawa campus, Waseda University (35° N latitude). None of the participants had been diagnosed with cardiac disease, diabetes, or chronic renal failure. We excluded participants who were on lipid and glucose lowering medications, or medications that could affect the study variables (*i.e.*, vitamin D supplements, vitamin D analogues, calcium, or any drugs that could affect bone and mineral metabolism, including bisphosphonates). We also recorded medication use including antihypertensive drugs that potentially affect glucose metabolism; 21 participants (19.6%) were treated with antihypertensive drugs. Current/former smoking status was assessed by a questionnaire. Daily alcohol and vitamin D intake were assessed using a brief-type self-administered diet history questionnaire [23]. All participants provided written informed consent before enrollment in the study, which was approved by the Ethical Committee of Waseda University. The study was conducted in accordance with the Declaration of Helsinki.

### 2.2. Anthropometric Characteristics

Body weight was measured by an electronic scale (Inner Scan BC-600, Tanita Inc., Tokyo, Japan), whereas height was measured by a stadiometer (YL-65, Yagami Inc., Nagoya, Japan). BMI was calculated from measurements of body weight and height. Visceral fat area (VFA) and subcutaneous fat area (SFA) were measured by magnetic resonance imaging (Signa 1.5T, General Electric Inc., Milwaukee, WI, USA). The imaging conditions included a T1-weighted spin-echo and axial-plane sequence with a slice thickness of 10 mm, repetition time of 140 ms, and echo time of 12.3 ms [22]. Cross-sectional images were scanned at the umbilical region. During the scan, the participants were asked to hold their breath for approximately 30 s after inhaling to reduce respiratory motion artifacts. Magnetic resonance images were transferred to a personal computer in the Digital Imaging and Communications in Medicine (DICOM) file format, and the cross-sectional VFA at the umbilical region was determined using image-analysis software (Slice-o-matic 4.3 for Windows, Tomovision, Montreal, QC, Canada). To minimize interobserver variation, all analyses were performed by the same investigator; the coefficient of variation was 0.4% for the cross-sectional areas of the umbilical region.

### 2.3. Cardiorespiratory Fitness

CRF was assessed by a maximal graded exercise test using a cycle ergometer (Ergomedic 828E; Monark, Varberg, Sweden) and quantified as maximal oxygen uptake ($\dot{\text{V}}$O_2_max). The graded cycle exercise began at a workload of 45–90 W, which was increased by 15 W/min until the subject could no longer maintain the required pedaling frequency of 60 rpm. Heart rate and ratings of perceived exertion were monitored each minute during exercise. During the incremental portion of the exercise test, expired gas was collected from the participants. O_2_ and CO_2_ concentrations were measured and averaged over 30 s intervals by an automated gas analyzer (Aeromonitor AE-300; Minato Medical Science, Tokyo, Japan). The maximum $\dot{\text{V}}$O_2_ recorded during the exercise test was considered the $\dot{\text{V}}$O_2_max (mL/kg/min), and the achievement of $\dot{\text{V}}$O_2_max was accepted if at least 3 of the following 4 criteria were met: the $\dot{\text{V}}$O_2_ curve showed a plateau despite increasing the work rate, maximal heart rate was 95% of the age-predicted maximal heart rate (220-age (in years)), respiratory exchange ratio >1.1, and perceived exertion ≥18. Participants were subsequently divided into the low and high CRF groups according to the median $\dot{\text{V}}$O_2_max value of each age group (mL/kg/min): 37.1 for 40–49 years, 38.8 for 50–59 years, 31.3 for 60–69 years, and 27.7 for 70–79 years.

### 2.4. Blood Sample Collection and Analysis

Blood samples were collected between 08:30 and 11:00 AM by accredited nurses or doctors after a 12-h overnight fast, and centrifuged at 3000× *g* for 15 min at 4 °C. Glucose, insulin, glycated hemoglobin (HbA1c), total cholesterol, high-density lipoprotein (HDL) cholesterol, low-density lipoprotein (LDL) cholesterol, and triglyceride concentrations were directly determined from fresh blood samples by BML Inc. (Tokyo, Japan). The HOMA-IR value was used as an index of insulin resistance; it was calculated from the fasting concentrations of plasma glucose and serum insulin as follows:

HOMA-IR = [fasting glucose (mg/dL)] × [fasting insulin (μU/mL)]/405
(1)

Serum 25(OH)D concentration was measured in duplicate using commercially available enzyme-linked immunosorbent assay kits (25(OH)D: Immundiagnostik AG, Bensheim, Germany) according to the manufacturer’s instructions. The intra- and interassay coefficients of variation were 8.9% and 10.6% for 25(OH)D. We divided participants into low and high 25(OH)D groups according to the median values of 25(OH)D concentration (36.3 nmol/L).

### 2.5. Statistical Analysis

All statistical analyses were performed using SPSS version 22.0 (SPSS, Inc., Chicago, IL, USA). Kolmogorov-Smirnov test was performed to assess the normality of data distribution, and several variables were log-transformed or square root transformed to obtain a normal distribution prior to analysis. Student’s *t*-test (for normal distributed variables), Mann-Whitney *U*-test (for non-normally distributed variables), or chi-square test (for categorical variables) was used to evaluate the significance of differences between the low and high CRF groups. Partial correlation analysis adjusted for age, season or VFA was performed to determine the associations between 25(OH)D concentration and subject characteristics. The influence of CRF levels and 25(OH)D concentration on blood parameters was evaluated by two-way analysis of covariance (ANCOVA) adjusted for the appropriate covariates. A post hoc test with Bonferroni correction was used to identify significant differences if a significant main effect or interaction was identified. Fisher’s exact test was used for comparing proportions of participants with high risk of insulin resistance (HOMA-IR ≥ 1.6) between combination groups. For this analysis, we created 4 subgroups for combinations of 25(OH)D and CRF on the basis of dichotomizing groups with low and high levels of each variable (High CRF and 25(OH)D subgroup, HH; High CRF and Low 25(OH)D subgroup, HL; Low CRF and High 25(OH)D subgroup, LH; Low CRF and 25(OH)D subgroup, LL). All measurements and calculated values are presented as mean (SD) (for normally distributed variables) or median (interquartile ratio; IQR) (for non-normally distributed variables) unless otherwise indicated. The level of significance was set at *p* < 0.05.

## 3. Results

The characteristics of study participants are shown in Table 1. The median 25(OH)D concentration was 36.3 (IQR: 26.4–50.2) nmol/L, 74.8% of participants were 25(OH)D deficient (<50 nmol/L), and 13.1% of participants had insufficient 25(OH)D (50–75 nmol/L). The median age of participants was 67.0 years for the low CRF group and 65.0 years for the high CRF group. The low CRF group had lower CRF, HDL cholesterol, 25(OH)D concentration, and vitamin D intake values and higher VFA, HbA1c, insulin and HOMA-IR values (*p* < 0.05) than the high CRF group. In addition, the low CRF group had slightly higher triglyceride levels than the high CRF group but was not statistically significant (*p* = 0.052).

**Table 1:** Subject characteristics.

Variable	All		Low CRF		High CRF		*p*
	(*n* = 107)		(*n* = 53)		(*n* = 54)		
Age (years)	65.0	(61.0–70.0)	67.0	(61.5–71.0)	65.0	(60.8–70.0)	0.300
Height (cm)	168.8	(6.5)	167.6	(5.7)	170.0	(7.1)	0.057
Weight (kg)	66.8	(8.7)	66.4	(8.8)	67.1	(8.7)	0.684
BMI (kg/m^2^)	23.4	(2.3)	23.6	(2.4)	23.1	(2.1)	0.328
VFA (cm^2^)	109.5	(46.9)	119.4	(48.2)	99.8	(43.4)	0.029
SFA (cm^2^)	82.5	(58.5–120.9)	77.8	(58.8–127.3)	91.1	(56.1–113.7)	0.528
CRF (mL/kg/min)	32.3	(27.4–36.2)	27.4	(22.8–29.8)	35.4	(32.5–39.1)	<0.001
Glucose (mg/dL)	98.2	(9.6)	99.0	(11.3)	97.4	(7.6)	0.396
HbA1c (%)	5.0	(0.3)	5.1	(0.3)	4.9	(0.2)	0.032
Insulin (μU/mL)	5.0	(3.5–6.5)	5.8	(4.0–8.1)	4.3	(3.0–5.9)	0.002
HOMA-IR	1.14	(0.86–1.66)	1.37	(0.94–1.94)	1.04	(0.72–1.37)	0.002
Total cholesterol (mg/dL)	213.9	(32.9)	213.5	(34.0)	214.2	(32.1)	0.907
HDL cholesterol (mg/dL)	61.0	(52.0–68.0)	59.0	(50.0–65.0)	64.0	(54.0–70.0)	0.028
LDL cholesterol (mg/dL)	122.5	(30.3)	124.4	(32.5)	120.6	(28.2)	0.529
Triglycerides (mg/dL)	91.0	(65.0–119.0)	97.0	(67.5–139.5)	83.0	(62.0–104.3)	0.052
25(OH)D (nmol/L)	36.3	(26.4–50.2)	33.2	(22.0–38.8)	45.3	(29.6–66.8)	<0.001
Vitamin D intake (μg/day)	12.6	(8.9–18.8)	10.8	(8.5–16.9)	14.2	(9.3–24.5)	0.034
Alcohol consumption (g/day)	21.5	(8.8–42.2)	15.7	(0.8–36.1)	25.4	(9.8–49.3)	0.106
Smoking status (%)	51.4		60.4		42.6		0.066
Medication use (%)	29.0		28.3		29.6		0.880

Data are mean (SD) or median (IQR) values. BMI: body mass index; VFA: visceral fat area; SFA: subcutaneous fat area; CRF: cardiorespiratory fitness quantified as $\dot{\text{V}}$O_2_max; HbA1c: glycated hemoglobin; HOMA-IR: homeostasis model assessment of insulin resistance; HDL: high-density lipoprotein; LDL: low-density lipoprotein; 25(OH)D: 25-hydroxyvitamin D.

Table 2 shows the correlation between 25(OH)D concentration and other variables. Results showed that 25(OH)D concentration was positively correlated with HDL cholesterol, vitamin D intake and CRF, and negatively correlated with insulin, HOMA-IR, triglycerides, and VFA (*p* < 0.05), after adjusting for age and season. Moreover, the relationships persisted after further adjustment for VFA.

**Figure 1:**
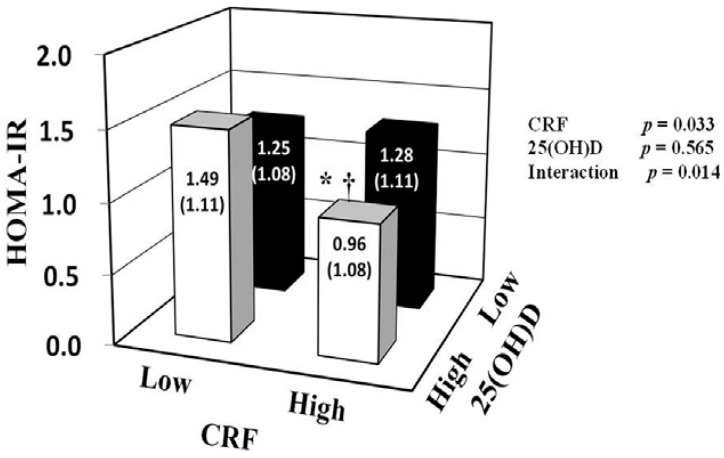
Associations of 25(OH)D and CRF with HOMA-IR. HOMA-IR was log transformed for two-way analysis of covariance with adjustment for age, season, visceral fat area, smoking status, medication use, vitamin D intake, and alcohol consumption. Data are shown as adjusted geometric mean ± SE. * *p* < 0.05 *vs.* low CRF within the same 25(OH)D group. ^†^ *p* < 0.05 *vs.* low 25(OH)D within the same CRF group. In the present study, 25(OH)D groups were divided according to the median serum 25(OH)D concentration (36.3 nmol/L), and CRF groups were divided according to the median $\dot{\text{V}}$O_2_max value of each age group (mL/kg/min): 37.1 for 40–49 years, 38.8 for 50–59 years, 31.3 for 60–69 years, and 27.7 for 70–79 years. CRF: cardiorespiratory fitness; HOMA-IR: homeostasis model assessment of insulin resistance; 25(OH)D: 25-hydroxyvitamin D.

To evaluate interaction effects between 25(OH)D and CRF on the blood parameters, two-way ANCOVA was performed after adjustment for potential confounders, including VFA (Table 3 and Figure 1). Significant interactions between 25(OH)D and CRF on insulin and HOMA-IR values were found (*p* < 0.05). No significant interactions were observed on glucose, HbA1c, triglycerides, and LDL cholesterol levels. We found significant main effects on insulin and HOMA-IR values for CRF (*p* = 0.034 and 0.033, respectively), but not for 25(OH)D. In the high CRF group (more than the median values of each age group), participants with high 25(OH)D concentration (≥36.3 nmol/L) had lower insulin (*p* = 0.038) and HOMA-IR (*p* = 0.043) values than participants with low 25(OH)D concentration (<36.3 nmol/L). In the high 25(OH)D group, participants with high CRF had lower insulin (*p* = 0.002) and HOMA-IR (*p* = 0.001) values than participants with low CRF. In addition, participants simultaneously in the high CRF and 25(OH)D subgroup (HH) had the lowest prevalence of insulin resistance (HOMA-IR ≥ 1.6) among all combination subgroups (8.6% for HH, 31.6% for HL, 47.4% for LH, 32.4% for LL, *p* < 0.05).

**Table 2:** Correlations of serum 25(OH)D with subject characteristics in middle-aged and elderly adults.

Variable	25(OH)D (Age- and Season-Adjusted)		25(OH)D (Age-, Season- and VFA-Adjusted)	
	*r*	*p*	*r*	*p*
BMI (kg/m^2^)	−0.033	0.742	0.127	0.198
VFA (cm^2^)	−0.199	0.042		
SFA (cm^2^)	0.022	0.822	0.152	0.124
CRF (mL/kg/min)	0.370	<0.001	0.340	<0.001
Glucose (mg/dL)	−0.009	0.929	0.000	0.999
HbA1c (%)	−0.134	0.171	−0.114	0.249
Insulin (μU/mL)	−0.348	<0.001	−0.301	0.002
HOMA-IR	−0.331	0.001	−0.283	0.004
Total cholesterol (mg/dL)	−0.015	0.879	−0.027	0.782
HDL cholesterol (mg/dL)	0.247	0.011	0.198	0.044
LDL cholesterol (mg/dL)	−0.087	0.375	−0.081	0.416
Triglycerides (mg/dL)	−0.304	0.002	−0.251	0.010
Vitamin D intake (μg/day)	0.299	0.002	0.280	0.004

BMI: body mass index; VFA: visceral fat area; SFA: subcutaneous fat area; CRF: cardiorespiratory fitness quantified as $\dot{\text{V}}$O_2_max; HbA1c: glycated hemoglobin; HOMA-IR: homeostasis model assessment of insulin resistance; HDL: high-density lipoprotein; LDL: low-density lipoprotein; 25(OH)D: 25-hydroxyvitamin D. Partial Pearson's correlation coefficients were calculated. SFA, insulin, HOMA-IR, HDL cholesterol, triglycerides, and vitamin D intake were log-transformed; 25(OH)D was square root transformed for analysis.

**Table 3:** Joint association of 25(OH)D and CRF with parameters related to insulin resistance.

Blood Variable	Low CRF		High CRF		CRF	25(OH)D	Interaction
	Low 25(OH)D	High 25(OH)D	Low 25(OH)D	High 25(OH)D	*p*	*p*	*p*
	(*n* = 34)	(*n* = 19)	(*n* = 19)	(*n* = 35)			
Glucose (mg/dL)	98.1 ± 1.6	100.6 ± 2.2	98.2 ± 2.3	97.1 ± 1.7	0.409	0.743	0.343
HbA1c (%)	5.06 ± 0.05	5.06 ± 0.06	5.02 ± 0.07	4.92 ± 0.05	0.115	0.433	0.340
Insulin (μU/mL)	5.2 ± 1.08	6.03 ± 1.1	5.3 ± 1.11	4.02 ± 1.08 *^,†^	0.034	0.494	0.016
HOMA-IR	1.25 ± 1.08	1.49 ± 1.11	1.28 ± 1.11	0.96 ± 1.08 *^,†^	0.033	0.565	0.014
Triglycerides (mg/dL)	103.5 ± 1.1	84 ± 1.1	88.9 ± 1.1	84.5 ± 1.1	0.458	0.200	0.403
LDL cholesterol (mg/dL)	126.9 ± 5.4	120.7 ± 7.2	118 ± 7.5	121.5 ± 5.5	0.541	0.842	0.442
HDL cholesterol (mg/dL)	60.3 ± 1	56.9 ± 1.1	57.8 ± 1.1	64.6 ± 1	0.368	0.582	0.058

CRF: cardiorespiratory fitness quantified as $\dot{\text{V}}$O_2_max; HbA1c: glycated hemoglobin; HOMA-IR: homeostasis model assessment of insulin resistance; HDL: high-density lipoprotein; LDL: low-density lipoprotein; 25(OH)D: 25-hydroxyvitamin D. Data are presented as the adjusted mean ± SE. Insulin, HOMA-IR, triglycerides and HDL cholesterol were log transformed (data are shown as adjusted geometric mean ± SE). Data were analyzed using two-way analysis of covariance (ANCOVA) adjusted for age, season, VFA, smoking status, medication use, vitamin D intake, and alcohol consumption. * *p* < 0.05 *vs.* low CRF within the same 25(OH)D group. ^†^ *p* < 0.05 *vs.* low 25(OH)D within the same CRF group. In the present study, 25(OH)D groups were divided according to the median serum 25(OH)D concentration (36.3 nmol/L) and CRF groups were divided according to the median $\dot{\text{V}}$O_2_max value of each age group (mL/kg/min): 37.1 for 40–49 years, 38.8 for 50–59 years, 31.3 for 60–69 years, and 27.7 for 70–79 years.

## 4. Discussion

To our knowledge, this study was the first to examine the combined associations of 25(OH)D and CRF with glucose metabolism in middle-aged and elderly Japanese men. Our results indicate that higher 25(OH)D concentration and CRF levels were associated with low risk of insulin resistance, independent of VFA levels. Furthermore, among participants with high CRF levels, higher serum 25(OH)D concentration largely strengthened the effect of CRF on reducing insulin resistance.

Several studies have shown that high levels of CRF are associated with low insulin resistance or incidence of T2DM in healthy adults including Japanese [19,20,21,22,24]. Usui *et al.* [22] revealed that fasting insulin concentrations and HOMA-IR were significantly lower among the high CRF group than the low CRF group in healthy Japanese women and men. Moreover, data from the Tokyo Gas Company in Japan showed that high CRF groups have significantly lower risk of developing T2DM compared with other fitness groups, even after controlling for BMI in healthy Japanese men [21,24]. Although previous studies have considered obesity levels using BMI, recent studies have revealed that reductions in the VFA may occur in the absence of changes in BMI [25]. The present study clearly showed that participants with higher CRF had significantly lower HOMA-IR and insulin values, independent of VFA levels, which is a more robust measure of obesity than the simple anthropometric measurement BMI. It indicates that high levels of CRF are critical for attenuating insulin resistance in middle-aged and elderly Japanese men, regardless of visceral obesity.

It has been reported that higher 25(OH)D concentration is strongly associated with low insulin and insulin resistance in adults [13,14]. However, those previous studies have been mostly limited to non-Asian populations. Furthermore, in place of directly measured CRF, self-reported physical activity determined by simple questionnaires was used as a controlled factor to determine the relationship between 25(OH)D concentration and HOMA-IR in those previous studies [7]. Although self-reported physical activity is related to CRF, prior evidence has demonstrated that CRF is much more strongly related with various health outcomes than self-reported physical activity, which often inevitably produces greater misclassification than the directly measured CRF [26,27,28]. In addition, the combined associations of serum 25(OH)D and CRF with glucose metabolism has not been examined. Consistent with the previous study [13,21,22], the present study revealed that higher 25(OH)D concentration and CRF levels were related to lower levels of fasting insulin and insulin resistance, independent of obesity status (VFA levels), in middle-aged and elderly Japanese men. Additionally, the present study is the first to demonstrate an interaction effect of serum 25(OH)D and CRF on glucose metabolism in Japanese middle-aged and elderly men. We found the relationship between 25(OH)D concentration and HOMA-IR was significantly affected by CRF levels. This was not surprising since 25(OH)D was more closely related with CRF than VFA levels (*r* = 0.370 *vs.* *r* = 0.199). The positive association between 25(OH)D and CRF could be explained partly by the variations in daily physical activity, sunlight exposure time, and cardiac morphology [29,30]. Moreover, we found that the values of HOMA-IR and the number of participants with high risk of insulin resistance were lower in the combined higher 25(OH)D and CRF group than in the other groups (*p* < 0.05). These observations suggest that the combination of higher CRF and 25(OH)D levels are probably more effective to reduce the risk of insulin resistance than either alone.

Although the clear mechanism is not well understood, several explanations can be given for this finding. Human and animal studies have reported that both higher levels of 25(OH)D and regular physical activity increased insulin secretion and enhanced insulin sensitivity in pancreatic and peripheral tissues [12,18,31]; thus, a combined effect of higher levels of 25(OH)D and CRF on glycemic control may be stronger than either alone. Additionally, it has been demonstrated that altered cholesterol metabolism (for example, low HDL cholesterol or high total cholesterol levels) may contribute to prevalence of insulin resistance [32], and regular physical activity and higher 25(OH)D concentration may improve lipid metabolism, resulting in low risk of insulin resistance [7,18,33]. Consistent with previous studies, the present study found that 25(OH)D and CRF are positively related to HDL cholesterol (*p* < 0.05 and *p* < 0.01, respectively), independent of age, season and VFA levels. Additionally, the two-way ANCOVA showed that participants simultaneously in the higher CRF and 25(OH)D group had higher HDL cholesterol levels than other groups, though not statistically significant (*p* = 0.06). Thus, it seems reasonable to infer that the combined effect of higher levels of 25(OH)D and CRF on glucose control is more effective than either alone, at least in our study population.

The present study has several limitations. First, because this was a cross-sectional study, it is difficult to make causal inferences between exposures and outcomes. A prospective study would provide more accurate associations of 25(OH)D concentration and CRF levels with insulin resistance. Second, the present study evaluated insulin resistance on the basis of HOMA-IR, which uses fasting values for estimation and mainly reflects insulin resistance in the liver [34]. Although HOMA-IR has been shown to have a good relationship with the hyperinsulinemic euglycemic clamp technique [35], a more accurate method for assessing insulin resistance, such as the oral glucose tolerance test or the glycemic insulin clamp test, should be used in future studies. Third, despite individuals had a higher daily vitamin D intake (12.6 μg/day) than Dietary Reference Intake (5.5 μg/day) for Japanese in the present study, the prevalence of 25(OH)D deficiency (74.8%) is still high; therefore, whether the findings can be extrapolated to reflect serum 25(OH)D sufficiency needs to be investigated. Finally, because we only examined men in the present study, our results should be interpreted with caution and confirmed in further cohorts of women. Despite these limitations, our study was the first to evaluate the combined associations of serum 25(OH)D concentration and CRF levels with insulin resistance, controlling for potential confounding factors including a precise obesity indicator in middle-aged and elderly Japanese men.

## 5. Conclusions

In conclusion, the present study revealed that higher 25(OH)D concentration and CRF levels were negatively correlated with levels of fasting insulin and insulin resistance in middle-aged and elderly Japanese men. These associations were independent of abdominal fat, while association between serum 25(OH)D and insulin resistance was largely influenced by CRF levels. Among middle-aged and elderly Japanese men with high levels of CRF, higher 25(OH)D concentration largely enhanced the effect of CRF on reducing insulin resistance. These results suggest that the combination of increasing both CRF and circulating 25(OH)D may be more effective for improving glycemic control than either alone.
